# Chronic obstructive pulmonary disease among former United States Department of Energy workers: comorbidities and lung function changes

**DOI:** 10.7717/peerj.20696

**Published:** 2026-02-03

**Authors:** Sara Howard, Louis Rocconi, Agricola Odoi

**Affiliations:** 1 Epidemiology and Exposure Science, Oak Ridge Associated Universities, Oak Ridge, TN, United States of America; 2 Biomedical and Diagnostic Sciences, University of Tennessee, Knoxville, TN, United States of America; 3 Evaluation, Statistics, & Methodology Program, Educational Leadership and Policy Studies Department, the University of Tennessee, Knoxville, TN, United States of America

**Keywords:** Chronic obstructive pulmonary disease, COPD, United states department of energy, Occupational exposure, National supplemental screening program, NSSP, Lung function changes, Hierarchical clustering, Random forest model, Comorbidity

## Abstract

**Background:**

Chronic Obstructive Pulmonary Disease (COPD) is a major cause of morbidity and mortality in the United States and is frequently associated with multiple comorbidities which lead to poor COPD outcomes in the general population. However, little is known regarding COPD comorbidities in occupational cohorts whose exposure experiences could result in differences in comorbidities compared to the general population. These differences may also be important for assessing COPD outcomes such as lung function changes or decline. Therefore, the objectives of this study were to: (1) identify and describe clusters of COPD comorbidities among Department of Energy (DOE) former workers; (2) assess if the attributes of the identified clusters differ from those identified among the general population based on the published literature, and (3) identify predictors of lung function changes and decline among DOE former workers.

**Methods:**

Clinical, occupational, and sociodemographic data were obtained from the National Supplemental Screening Program. Imputation for missing values was performed using multiple imputation by chained equation. Comorbidity clusters were identified using hierarchical clustering. Regression and classification random forest models were used to identify predictors of changes in forced expiratory volume in one second (FEV_1_) and FEV_1_ decline. Variable importance scores were used to assess the predictive importance of the predictors.

**Results:**

A total of 17,448 DOE former workers were included in this study, 20.9% of whom had COPD at their initial exam. Four comorbidity clusters were identified among those with COPD. Cluster 1 was composed of individuals with low prevalence of comorbidities, cluster 2 contained individuals with high prevalence of cardiovascular diseases, cluster 3 had those with high lung cancer prevalence, while cluster 4 had individuals with high prevalence of multiple comorbidities. There was no significant association between the clusters and either FEV_1_ change or decline. Age at hire, welding fume exposure, and silica exposure were significant predictors of both FEV_1_ changes and decline. Age at initial exam and baseline FEV_1,_ which have been identified as significant predictors of these outcomes in the general population, were also significantly associated with the outcomes in the current study. By contrast, smoking, which is a common risk factor in the general population, was a weak predictor of FEV_1_ change and decline in this cohort.

**Conclusion:**

Clusters of COPD related comorbidities were identified. The most important predictors of lung function changes and decline were FEV_1_, age, age at hire, and sex. The findings suggest that the important predictors of lung function changes and decline in this occupational cohort are different from those reported in the general population. Study findings may be useful for guiding enhanced monitoring efforts and control programs.

## Background

Chronic Obstructive Pulmonary Disease (COPD), a chronic inflammatory disease of the lungs, is a major cause of morbidity and mortality in the United States (US) and is frequently associated with multiple comorbidities (Syamlal et al., 2020; Smith & Wrobel, 2014; Wheaton et al., 2019). Approximately 80% of individuals with COPD have at least one comorbidity (Corlateanu et al., 2016). Moreover, the prevalence and number of comorbidities tend to increase with age (Alter et al., 2022). The mean number of comorbidities is estimated to be as high as five conditions, although this varies based on the population (Fabbri et al., 2023). The most commonly reported comorbidities in the general population include multiple cardiovascular outcomes, stroke, hypertension, hyperlipidemia, diabetes, metabolic syndrome, chronic kidney disease, pulmonary and extra-pulmonary cancers, cachexia, skeletal muscle wasting, osteoporosis, gastro-esophageal reflux, anemia, arthritis, depression, sleep apnea, and other respiratory non-malignant respiratory diseases (Barr et al., 2009; Barnes & Celli, 2009; Cazzola et al., 2010; Negewo, Gibson & McDonald, 2015; Corlateanu et al., 2016; Fabbri et al., 2023). The presence of these comorbid conditions is associated with poor health outcomes including lower quality of life as well as higher rates of dyspnea, hospitalizations, and mortality (Sin et al., 2006; Vestbo et al., 2011; Tantucci & Modina, 2012; Smith & Wrobel, 2014; Hillas et al., 2015).

In clinical practice, comorbidities are important considerations when making disease management decisions (Barnes & Celli, 2009; Hillas et al., 2015). Therefore, identifying common combinations or clusters of comorbidities and assessing if these disease combinations impact health outcomes is critically important for guiding clinical decisions and case management for improving health outcomes. Significant efforts have been taken to identify comorbid clusters in the general population as well as their impact on disease progression (Vanfleteren et al., 2013; Miller et al., 2013; Burgel, Paillasseur & Roche, 2014; Rennard et al., 2015; Chubachi et al., 2016). Comorbidity clusters identified among the general population typically include a combination of generally healthy, cardiovascular disease, metabolic conditions, and psychological conditions clusters (Vanfleteren et al., 2013; Chubachi et al., 2016; Jureviciene et al., 2022; Vikjord et al., 2022; James et al., 2024). However, information on COPD comorbidities in occupational cohorts is limited. These comorbidities may differ from those in the general population due to differences in exposure experiences related to occupations. These differences, if identified, may be important for guiding clinical management. This is particularly critical among Department of Energy (DOE) workers who historically were exposed to respiratory hazards unique to the work they performed within the DOE complex. These respiratory hazards, which could increase their risk of COPD, include: cadmium, diesel exhaust, welding fumes, and silica (Hart, Eisen & Laden, 2012; Möhner, Kersten & Gellissen, 2013; Oh et al., 2014; Koh et al., 2015). It is also possible that the effect of these comorbidities on the progression of COPD may differ in this occupational cohort compared to the general population. Thus, further investigation of COPD related comorbidities in this occupational cohort is warranted to better guide clinical decisions, disease management, and improvement of health outcomes. Therefore, the objectives of this study were to: (1) identify and describe clusters of COPD comorbidities among Department of Energy (DOE) former workers; (2) assess if the attributes of the identified clusters differ from those identified among the general population based on the published literature, and (3) identify predictors of lung function changes and decline among DOE former workers.

## Methods

### Ethical statement

The United States Central Department of Energy Institutional Review Board (IRB) provided ethics approval and oversight of the study (DOE ID: DOE000645). A waiver of informed consent was granted by the IRB because this was a retrospective study involving analysis of secondary data.

### Study population

Data for this study were provided by the National Supplemental Screening Program (NSSP), which is a free medical screening program for DOE former workers, contractors, and subcontractors in the US. This voluntary program screens participants for occupational illnesses as well as general health conditions at facilities across the United States. The initial examination occurs when DOE workers leave employment at the department. After the initial examination, they are eligible for rescreening every three years. At both the initial and follow-up screening exams, participants are offered a wide range of clinical tests tailored to their self-reported occupational exposures, work histories, and medical histories. These clinical tests included metabolic panels, spirometry, beryllium lymphocyte proliferation test (BeLPT), and chest X-rays with International Labor Organization (ILO) B-reading. The protocol used was developed by physicians and reviewed every two years. Supplementary guidance is also provided by the United States (US) Preventative Services Task Force, and the National Institution for Occupational Safety and Health (NIOSH) Total Worker Health frameworks. All participants may opt out of any of the suggested clinical tests (Stange et al., 2016).

Since the NSSP is voluntary, participants may choose to participate at any time post-employment. Similarly, they may choose to extend the time between exams for longer than three years (Stange et al., 2016; United States Department of Energy, 2019). Eligibility for this study required participants to complete their initial exam, including spirometry results, before January 1, 2020 (*n* = 18,075). A subset of participants (*n* = 587) was excluded from the study due to their self-reported medical history of restrictive lung diseases such as chronic beryllium disease, silicosis, or asbestosis as these conditions may present with obstructive lung function patterns, potentially leading to misclassification as COPD (*i.e.,* false positive) (Martinez-Pitre, Sabbula & Cascella, 2023). For individuals with repeated clinical tests, only the results from the initial and first rescreen exam were assessed in this study. The final full cohort for this study had 17,488 participants.

### Data sources and preparation

#### Chronic obstructive pulmonary disease definition and data

Data for this study were provided by the US DOE’s National Supplemental Screening Program (NSSP), which is a free medical screening program for former DOE workers, contractors, and subcontractors in the US. This voluntary program screens participants for occupational illnesses as well as general health conditions. Participants are eligible for rescreening every three years (Stange et al., 2016; United States Department of Energy, 2019). Annually, the NSSP conducts approximately 2,000 screening exams, which include both initial and follow-up exams (Stange et al., 2016).

COPD was classified using the Global Initiative for Chronic Obstructive Lung Disease (GOLD) criterion, which compares the ratio of forced expiratory volume in one second (FEV_1_) to forced vital capacity (FVC) with a fixed standard of 0.7 (Global Initiative for Chronic Obstructive Lung Disease, 2020). If the FEV_1_/FVC ratio was less than the fixed standard (*i.e.,* FEV_1_/FVC < 0.7), then the individual was considered to have COPD (Global Initiative for Chronic Obstructive Lung Disease, 2020). For descriptive purposes, COPD severity was also categorized as mild (FEV_1_ ≥ 80% of predicted), moderate (FEV_1_ 50–79% of predicted), severe (FEV_1_ 30–49% of predicted), and very severe (FEV_1_ < 30% of predicted) (Bakke et al., 2011; Global Initiative for Chronic Obstructive Lung Disease, 2020). Previous history of COPD was not included as part of the COPD classification for this study.

#### Comorbidities data

The comorbid conditions considered for investigation were identified from the scientific literature and the data extracted from participants’ self-reported medical histories. The conditions assessed included: myocardial infarction, coronary artery disease, hypertension, stroke, underweight, overweight or obese, kidney problems, rheumatoid arthritis, asthma, diabetes, lung cancer, and non-pulmonary cancer. Participants who reported a diagnosis of diabetes or who reported routine insulin use were classified as having a history of diabetes. Additionally, participants who reported any indication of cancer occurring outside of the lungs were classified as having non-pulmonary cancers. The remaining comorbidities were extracted as binary indicators (Yes/No) from the medical histories (Table 1).

**Table 1:** Variables investigated for potential association with lung function changes.

**Variable**	**Variable definition**
**Demographic, Clinical, & Behavioral**	
Age at Initial Exam (in Years)	Age of study participant at initial exam
Time between Exams (in Years)	Time from initial exam to rescreen exam
^1^FEV_1_ at Initial Exam	Captured at initial exam
Race	Classified as black or non-Black
Sex	Categorized as male or female
Smoking Status	Self-reported at initial exam & classified as smoker/never smoker
**Occupational History**	
^2^DOE Site Category	Classified as uranium processing, weapons production, and science and laboratory facilities
Age at Hire (in Years)	Age of study participant at self-reported hire date
Duration of Employment	Self-reported and categorized into 5-year categories
Cadmium Exposure	Self-reported at initial exam (Yes/No)
Silica Exposure	Self-reported at initial exam (Yes/No)
Diesel Exhaust Exposure	Self-reported at initial exam (Yes/No)
Welding fumes exposure	Self-reported at initial exam (Yes/No)
**Respiratory Symptoms**	
Frequent cough	Self-reported at initial exam (Yes/No)
Shortness of breath	Self-reported at initial exam (Yes/No)
Wheezing	Self-reported at initial exam (Yes/No)
Other breathing problems	Self-reported at initial exam (Yes/No)
**Medical History**	
Comorbidity Cluster	Assigned after hierarchical clustering (Clusters 1-4)
Myocardial Infarction	Self-reported at initial exam (Yes/No)
Coronary Artery Disease	Self-reported at initial exam (Yes/No)
Hypertension	Self-reported at initial exam (Yes/No)
Stroke	Self-reported at initial exam (Yes/No)
Underweight	Self-reported at initial exam (Yes/No)
Overweight or Obese	Self-reported at initial exam (Yes/No)
Kidney Problems	Self-reported at initial exam (Yes/No)
Rheumatoid Arthritis	Self-reported at initial exam (Yes/No)
Lung Cancer	Self-reported at initial exam (Yes/No)
Non-Pulmonary Cancer	Self-reported at initial exam (Yes/No)
Asthma	Self-reported at initial exam (Yes/No)

**Notes.**

1 Forced expiratory volume in one second is abbreviated as FEV_1_.

2 Department of Energy is abbreviated as DOE.

#### Demographic, clinical, behavioral, and work history Data

The demographic, clinical, and behavioral factors assessed in this study included age at initial exam, self-reported respiratory symptoms, FEV_1_ value at initial exam, time between exams, sex (male/female), race (Black/non-Black), and self-reported smoking status (smoker/never smoker). For the occupational histories, the type of DOE facility where the participant spent the majority of their career was classified by the NSSP as uranium processing, weapons production, or science and laboratory facility. Duration of employment at a DOE facility was calculated from self-reported hire and termination dates. Age at hire was also computed from the self-reported hire dates and birth dates. The occupational exposures to silica, diesel exhaust, welding fumes, and cadmium were also self-reported and captured as dichotomous (Yes/No) variables.

The occupational hazards that DOE workers may be exposed to vary widely depending on job roles. However, the Former Worker Program (FWP) medical protocol, used in the study, collected data on the following occupational hazards: asbestos, beryllium, plutonium and other ionization radiation sources, silica, diesel exhaust, welding fumes, cadmium, resins, solvents, nickel, lead, mercury, benzene, formaldehyde, and noise (Department of Energy, 2022).

### Statistical & machine learning analyses

#### Descriptive statistics

All data management, statistical analyses, and machine learning were performed in SAS 9.4 (SAS Institute, 2016) and RStudio version 2021.09.2 (RStudio Team, 2020) interface of R version 4.0.3 (R Core Team, 2020). Basic descriptive analyses for categorical variables included computation of frequencies and percentages as well as 95% confidence intervals of the percentages. Continuous variables were assessed for normality of distribution using the one-sample Kolmogorov–Smirnov test and a critical *p*-value of 0.05 (Massey, 1951). Since most of the continuous variables were not normally distributed, summary statistics were presented as quartiles.

#### Multiple imputation

Imputation of missing values was performed using multivariate imputation by chained equation (MICE) through the mice R package (Van Buuren & Groothuis-Oudshoorn, 2011). Through this process, imputation was performed using predictive mean matching with a maximum of 30 iterations for 20 total imputations across all variables considered for comorbidity cluster identification as well as investigation of predictors of lung function changes (Sterne et al., 2009).

#### Investigation of chronic obstructive pulmonary disease comorbidity clusters

Before performing the cluster analysis, univariable logistic regression models were used to identify comorbid conditions in the imputed datasets that had significant associations with COPD using a relaxed alpha of 0.2, and the results were combined using Rubin’s rules for pooling (Rubin, 1987). Only comorbid conditions that had univariable logistic regression *p* ≤ 0.2 were included in the cluster analysis.

To identify comorbidity clusters specific to COPD, hierarchical clustering using an agglomerative approach was applied only to participants with COPD. The cluster analysis used the complete linkage strategy for cluster merging and Gower’s distance (Gower, 1971) as the distance measure. The multiple imputation pooling framework developed by Basagaña et al. (2013) was applied to the hierarchical clustering with the MICE datasets. In this approach, Basagaña et al. (2013) propose performing hierarchical cluster analysis on each imputed dataset individually to identify the optimal number of clusters for each dataset using an accepted method of optimizing the number of clusters. Then, Basagaña et al. (2013) suggest using the median or mode of those values to find the overall optimal number of clusters across all the imputed datasets. In this study, the “elbow” method was chosen for determining the optimal number of clusters in each imputed dataset. The point at which the smallest decrease in the within cluster sum of squares occurs, creating an elbow on the graph, indicates the optimal number of clusters. After performing the elbow method for each data, the median of those optimal number of clusters was chosen for the overall optimal number of clusters. The imputed datasets were re-analyzed using the overall optimal number of clusters. Summary statistics on cluster membership and prevalence of comorbidities within each cluster were reported (Basagaña et al., 2013). Kruskal–Wallis, followed by Dunn’s Test with a Bonferroni correction, was used to determine if the percentages of comorbidities varied across clusters. All cluster analyses were performed using the cluster R package (Maechler et al., 2023).

#### Investigation of predictors of lung function changes and decline

Two approaches were used to predict lung function changes: a regression random forest model predicting the percent of FEV_1_ change at the rescreen exam and a classification random forest model predicting whether the FEV_1_ value declined at the rescreen exam. Random forest models were chosen due to their ability to analyze high dimensional data without overfitting while still providing the flexibility to predict both continuous and categorical variables (Hastie, Tibshirani & Friedman, 2009). First, the regression random forest model was used to identify variables that had a significant association with the percentage change in FEV_1_ using univariable ordinary least squares models and a relaxed alpha of 0.2. The results were then pooled using Rubin’s rules for pooling (Rubin, 1987), and only variables with a *p*-value ≤ 0.2 were included in the regression random forest. Each MICE dataset was split into 75% training data and 25% testing data. These data only included individuals with COPD who completed their initial exam and returned for their first rescreen exam. Model performance was assessed using root mean squared error. To identify which variables were most important for prediction, variable importance scores were calculated using the sum of squared errors. The variable importance scores indicate the usefulness of the factor for predicting each outcome. Since the model prediction was performed over each of the 20 MICE datasets, the median variable importance scores for each feature were presented.

As with the regression random forest models, the classification random forest models used univariable logistic regression with a relaxed alpha of 0.2 to identify variables associated with a decline in FEV_1_ at the rescreen exam. The logistic regression model results were pooled using Rubin’s Rules (Rubin, 1987). Only variables with a *p*-value ≤ 0.2 were included in the classification random forest models. As before, the MICE datasets were split into training data (75%) and testing data (25%). As with the regression random forest models, the training and testing data included only individuals with COPD who completed both an initial and rescreen exam. Model performance assessment was based on the overall classification accuracy as well as the sensitivity and specificity. Variable importance scores were once again used for identifying the most important factors driving model performance. However, this time the classification variable importance scores were calculated using the Gini impurity index to account for the binary outcome.

The full list of variables considered for both the regression and classification random forest models is provided in Table 1 and includes the comorbidity clusters identified through the cluster analysis. If the comorbidity clusters were not selected for the random forest models, then the individual comorbidity components of the clusters were considered for possible inclusion in the random forest models. Descriptive statistics (*i.e.,* median, minimum, maximum, interquartile range) were used to describe the performance metrics and variable importance scores across imputed datasets for both the regression and classification random forest models. All the random forest model building, variable selection, and descriptive statistics were performed using the caret R package (Kuhn, 2008).

## Results

### Descriptive statistics

A total of 17,488 NSSP participants completed their initial exams before 2020, and 20.9% of them had COPD (Table 2). The majority of COPD cases were mild (43.0%) or moderate (42.8%) and only 14.2% were severe or very severe. Most of the participants were male (74.7%) and non-black (88.2%). Approximately 43.6% of the participants reported a history of smoking. The median age at initial exam was 66 years old, and nearly 22% of the participants were 75 years old or older (Table 2).

**Table 2:** Descriptive statistics of work history, demographic, and clinical characteristics of Department of Energy former workers participating in the National Supplemental Screening Program, 2005–2019.

**Categorical characteristics**	**Frequency**	**Percentage**	**95% confidence interval**
**COPD**	***n***=**17,488**		
Yes	3,647	20.9	20.3–21.5
No	13,841	79.2	78.5–79.7
**GOLD level**	***n***=**3,647**		
Mild	1,569	43.0	41.4–44.6
Moderate	1,561	42.8	41.2–44.4
Severe or very severe	515	14.2	13.0–15.3
**Sex**	***n***=**17,488**		
Male	13,064	74.7	74.1–75.4
Female	4,424	25.3	24.7–25.9
**Race**	***n***=**13,525**		
Black	1,615	11.9	11.3–12.4
Non-Black	12,010	88.2	87.6–88.7
**Smoking Status**	***n***=**17,488**		
Ever Smoker	7,619	43.6	42.8–44.3
Never Smoker	9,869	56.4	55.7–57.2
**Age**	***n***=**17,488**		
<55 years old	3,122	17.9	17.3–18.4
55–59 years old	2,095	12.0	11.5–12.5
60–64 years old	2,882	16.5	15.9–17.0
65–69 years old	3,015	17.3	16.7–17.9
70–74 years old	2,586	14.8	14.3–15.3
≥ 75 years old	3,778	21.6	21.0–22.2
^1^ **DOE category**	***n***=**17,488**		
Weapons production	12,763	73.0	72.3–73.6
Science/Laboratory	3,600	20.6	20.0–21.2
Uranium processing	1,125	6.4	6.1–6.8
**Welding fumes exposure**	***n***=**17,488**		
Yes	8,221	47.1	46.4–47.9
No	9,227	52.9	52.1–53.6
**Cadmium exposure**	***n***=**17,455**		
Yes	6,206	35.6	34.8–36.3
No	11,249	64.5	63.7–65.2
**Silica exposure**	***n***=**17,452**		
Yes	6,077	34.8	34.1–35.5
No	11,375	65.2	64.4–65.9
**Diesel exhaust exposure**	***n***=**17,464**		
Yes	5,072	29.0	28.4–29.7
No	12,392	71.0	70.3–71.6
**Duration of employment**	***n***=**17,488**		
<5 years	5,605	32.1	31.4–32.8
5–9 years	2,886	16.5	16.0–17.1
10–14 years	2,008	11.5	11.0–12.0
15–19 years	1,551	8.9	8.5–9.3
20–24 years	1,375	7.9	7.5–8.3
25–29 years	1,229	7.0	6.7–7.4
≥ 30 years	2,834	16.2	15.7–16.8
**Shortness of breath**	***n***=**17,465**		
Yes	7,618	43.6	42.9–44.4
No	9,847	56.4	55.7–57.1
**Frequent cough**	***n***=**17,461**		
Yes	5,890	33.7	33.0–34.4
No	11,571	66.3	65.6–67.0
**Wheezing**	***n***=**17,437**		
Yes	4,643	26.6	26.0–27.3
No	12,794	73.4	72.7–74.0
**Other breathing problems**	***n***=**17,438**		
Yes	4,024	23.1	22.5–23.7
No	13,414	76.9	76.3–77.6
**Overweight or obese**	***n***=**17,445**		
Yes	13,900	79.7	79.1–80.3
No	3,545	20.3	19.7–20.9
**Hypertension**	***n***=**17,032**		
Yes	6,312	37.1	36.3–37.8
No	10,720	62.9	62.2–63.7
**Non-pulmonary cancer**	***n***=**17,345**		
Yes	4,900	28.3	27.6–28.9
No	12,445	71.7	71.1–72.4
**Diabetes**	***n***=**17,247**		
Yes	3,008	17.4	16.9–18.0
No	14,239	82.6	82.0–83.1
**Asthma**	***n***=**17,044**		
Yes	2,247	13.2	12.7–13.7
No	14,797	86.8	86.3–87.3
**Kidney problems**	***n***=**14,881**		
Yes	1,730	11.6	11.1–12.1
No	13,151	88.4	87.9–88.9
**Coronary artery disease**	***n***=**16,832**		
Yes	1,919	11.4	10.9–11.9
No	14,913	88.6	88.1–89.1
**Myocardial infarction**	***n***=**16,945**		
Yes	1,445	8.5	8.1–9.0
No	15,500	91.5	91.1–91.9
**Stroke**	***n***=**17,087**		
Yes	857	5.0	4.7–5.3
No	16,230	95.0	94.7–95.3
**Underweight**	***n***=**17,445**		
Yes	83	0.5	0.4–0.6
No	17,362	99.5	99.4–99.6
**Anemia**	***n***=**17,018**		
Yes	1,619	9.5	9.1–10.0
No	15,399	90.5	90.1–90.9
**Rheumatoid arthritis**	***n***=**15,836**		
Yes	932	5.9	5.5–6.3
No	14,904	94.1	93.8–94.5
**Lung cancer**	***n***=**17,087**		
Yes	135	0.8	0.7–0.9
No	16,952	99.2	99.1–99.3
**Continuous characteristics**	**Frequency**	**Median**	**Interquartile range**
Age at initial exam (Years)	17,488	66	58–73
Age at hire (Years)	17,453	26	22–34
^2^FEV_1_ at initial	17,471	2.75	2.17–3.35
^3^Time between exams (Years)	769	5	3–7
^2^^,^^3^FEV_1_ change between exams	769	−0.27	−1.06–0.43

**Notes.**

1 Department of Energy.

2 Forced expiratory volume in one second.

3 Computed for participants with chronic obstructive pulmonary disease who returned for a rescreen exam.

Seventy three percent of the participants worked at weapons production facilities, 20.6% at science or laboratory facilities, and 6.4% at uranium processing facilities. Exposure to welding fumes was the most prevalent occupational exposure affecting 47.1% of the participants followed by cadmium (35.6%), silica (34.8%), and diesel exhaust (29.0%) exposures. The median age at hire was 26 years (interquartile range (IQR): 22–34 years), with 32.1% of participants employed for less than 5 years at a Department of Energy (DOE) facility.

Regarding clinical presentation, participants most frequently (43.6%) reported shortness of breath (43.6%), followed by frequent cough (33.7%), wheezing (26.6%), and other breathing problems (23.1%). Overweight or obesity was the most prevalent comorbid condition observed in 79.7% of the participants followed by hypertension (37.1%), non-pulmonary cancer (28.3%), diabetes (17.4%), asthma (13.2%), kidney problems (11.6%), coronary artery disease (11.4%), and the remaining conditions had a prevalence of <10%. The median FEV_1_ at the initial exam was 2.8 (IQR: 2.2–3.4).

Only 22.3% of the participants returned for the first rescreening exam, and of those, only 19.7% (*n* = 769) were classified as having COPD at their initial exam. The median time between exams for those with COPD was 5 years (IQR: 3–7 years). Approximately 59% of those with COPD showed a decline in the FEV_1_ at their rescreen exam, and their median change in FEV_1_ from the initial to rescreen exam was −0.27 (Table 2). All assessed comorbidities, except diabetes, had significant associations with COPD at a liberal alpha level of 0.2 and were included in the hierarchical cluster analysis (Table 3).

**Table 3:** Associations between COPD 1 and several comorbidities among Department of Energy former workers who participated in the National Supplemental Screening Program, 2005–2019.

**Characteristics**	**Odds ratio**	**95% Confidence interval**	***p*-value**
**Lung cancer**			
Yes	3.94	2.82, 5.50	<0.0001
No	*Reference*		
**Myocardial infarction**			
Yes	1.36	1.21, 1.54	<0.0001
No	*Reference*		
**Coronary artery disease**			
Yes	1.43	1.28, 1.60	<0.0001
No	*Reference*		
**Hypertension**			
Yes	1.07	0.99, 1.16	0.0717
No	*Reference*		
**Stroke**			
Yes	1.52	1.30, 1.78	<0.0001
No	*Reference*		
**Diabetes**			
Yes	0.96	0.87, 1.05	0.3673
No	*Reference*		
**Underweight**			
Yes	3.23	2.09, 4.98	<0.0001
No	*Reference*		
**Overweight or obese**			
Yes	0.62	0.57, 0.67	<0.0001
No	*Reference*		
**Kidney problems**			
Yes	1.21	1.08, 1.36	0.0011
No	*Reference*		
**Rheumatoid arthritis**			
Yes	1.33	1.15, 1.54	0.0002
No	*Reference*		
**Anemia**			
Yes	1.08	0.96, 1.23	0.1978
No	*Reference*		
**Non-pulmonary cancer**			
Yes	1.35	1.24, 1.46	<0.0001
No	*Reference*		
**Asthma**			
Yes	1.92	1.74, 2.11	<0.0001
No	*Reference*		

**Notes.**

1 Chronic obstructive pulmonary disease.

### Comorbidity clusters

The overall optimal number of clusters was 4 with a range of 3 to 7 clusters. Cluster 1 was the largest with a median of 69.1% (IQR: 69.1%–77.6%) participants that had COPD across all datasets. Cluster 4 was the smallest and had a median of 2.1% (IQR:1.3%–4.7%) of the participants. Clusters 2 and 3 had medians of 21.4% (IQR: 12.1%–26.4%) and 4.9% (IQR: 2.3–7.3) of the participants, respectively.

The results of the Kruskal–Wallis test showed that the prevalence of nearly all comorbid conditions, except asthma and underweight, varied across clusters (Table 4). Although there was evidence that lung cancer prevalence varied across clusters based on the Kruskal–Wallis test (*p* = 0.0396), there was no evidence of variation across clusters after Bonferroni correction was applied to the Dunn’s test since all corrected *p*-values were >0.05 (Table 4). As for the conditions which did vary across clusters, the prevalence of myocardial infarction was significantly (*p* < 0.05) lower in cluster 1 (median: 5.3%) than in all other clusters (Table 4), while the prevalence in cluster 2 (median: 18.8%) was significantly (*p* = 0.0148) lower than that in cluster 4 (median: 56.0%). Similarly, coronary artery disease prevalence was significantly (*p* < 0.05) lower in cluster 1 (median: 6.3%) than all other clusters (Table 4). Cluster 2 (median: 29.1%) also had a significantly (*p* = 0.0240) lower prevalence of coronary artery disease than cluster 4 (median: 77.8%). The results of the comparisons of the prevalence estimates of the rest of the comorbid conditions across clusters are shown in Table 4. Generally, cluster 1 had low prevalence of most comorbidity conditions. Cluster 2 had higher prevalence proportions of cardiovascular conditions when compared to cluster 1 while also having lower prevalence proportions of most conditions than clusters 3 and 4. Both clusters 3 and 4 had high prevalence proportions of nearly all conditions.

**Table 4:** Summary of comorbidities within clusters of National Supplemental Screening Program participants with chronic obstructive pulmonary disease (*n* = 3, 647) across all imputed datasets (*n* = 20), 2005–2019.

Comorbidity	Cluster 1 Median (IQR^1^)	Cluster 2 Median (IQR^1^)	Cluster 3 Median (IQR^1^)	Cluster 4 Median (IQR^1^)	Kruskal– Wallis Test Statistic (*p*-value)	Clusters 1 & 2 Dunn’s Test Statistic^2^ (*p*-value)	Clusters 1 & 3 Dunn’s Test Statistic^2^ (*p*-value)	Clusters 1 & 4 Dunn’s Test Statistic^2^ (*p*-value)	Clusters 2 & 3 Dunn’s Test Statistic^2^ (*p*-value)	Clusters 2 & 4 Dunn’s Test Statistic^2^ (*p*-value)	Clusters 3 & 4 Dunn’s Test Statistic^2^ (*p*-value)	
Lung cancer	1.8 (1.4, 2.1)	2.3 (1.5, 4.1)	3.1 (1.8, 4.7)	1.3 (0.7, 2.2)	8.3 (0.0396)	1.45 (0.8721)	1.91 (0.3405)	−0.59 (1.000)	0.45 (1.000)	−2.04 (0.2473)	−2.49 (0.0765)	
Myocardial infarction	5.3 (3.4, 7.5)	18.8 (9.8, 32.7)	42.1 (12.5, 68.9)	56.0 (34.8, 80.3)	38.1 (<0.0001)	2.92 (0.0211)	4.31 (<0.0001)	5.95 (<0.0001)	1.39 (0.9907)	3.03 (0.0148)	1.64 (0.6063)	
Coronary artery disease	6.3 (3.2, 11.0)	29.1 (10.8, 49.2)	53.2 (16.8, 84.3)	77.8 (36.1, 89.6)	37.4 (<0.0001)	2.91(0.0215)	4.50 (<0.0001)	5.79 (<0.0001)	1.59(0.6773)	2.88(0.0240)	1.29(1.000)	
Hypertension	31.0 (20.2, 35.3)	66.8 (41.9, 76.0)	55.9 (34.6, 81.2)	80.1 (65.4, 93.6)	35.3 (<0.0001)	3.82 (0.0008)	3.64 (0.0016)	5.82 (<0.0001)	−0.17 (1.000)	2.00 (0.2705)	2.18 (0.1767)	
Stroke	4.9 (3.5, 5.9)	9.7 (5.4, 12.2)	18.3 (7.2, 40.3)	31.1 (12.8, 70.7)	27.6 (<0.0001)	2.25 (0.1458)	3.98 (0.0004)	4.87 (<0.0001)	1.73 (0.5036)	2.62 (0.0528)	0.89 (1.000)	
Kidney problems	10.3 (8.2, 12.6)	9.7 (5.3, 21.5)	36.3 (19.8, 54.2)	49.4 (39.0, 72.0)	42.1 (<0.0001)	0.67 (1.000)	4.06 (0.0003)	5.50 (<0.0001)	3.40 (0.0041)	4.84 (<0.0001)	1.44 (0.8950)	
Rheumatoid arthritis	5.4 (4.0, 7.3)	10.0 (3.5, 13.0)	25.5 (9.3, 33.4)	37.0 (15.5, 53.7)	41.3 (<0.0001)	1.76 (0.4681)	4.54 (<0.0001)	5.78 (<0.0001)	2.78 (0.0327)	4.02 (0.0004)	1.24 (1.000)	
Anemia	0.09 (0.08, 0.10)	0.07 (0.03, 0.15)	0.25 (0.19, 0.38)	0.33 (0.22, 0.53)	35.7 (<0.0001)	−0.12 (1.000)	3.56 (0.0022)	4.64 (<0.0001)	3.68 0.0014	4.75 (<0.0001)	1.08 (1.000)	
Non-Pulmonary Cancer	28.6 (24.4, 30.9)	48.7 (34.3, 56.3)	55.8 (44.2, 64.7)	60.5 (48.7, 76.9)	24.3 (<0.0001)	2.99 (0.0169)	4.21 (0.0002)	4.31 (<0.0001)	1.22 (1.000)	1.33 (1.000)	0.10 (1.000)	
Overweight or Obese	68.5 (65.0, 73.8)	85.0 (67.3, 94.3)	79.7 (62.4, 89.1)	81.3 (75.7, 89.9)	10.2 (0.0172)	2.76 (0.0351)	1.91 (0.3379)	2.77 (0.0341)	−0.85 (1.000)	0.01 (0.9919)	0.86 (0.3913)	
Underweight	1.1 (0.8, 1.4)	0.4 (0.1, 1.9)	0.4 (0.0, 1.7)	0.7 (0.0, 2.5)	2.2 (0.5320)						
Asthma	19.5 (17.1, 20.5)	16.3 (7.0, 24.0)	17.7 (11.5, 46.3)	25.3 (13.2, 57.9)	4.6 (0.205)						

**Notes.**

1 Interquartile range.

2 Dunn’s test was only performed on comorbidities which varied across clusters based on the Kruskal–Wallis test (*p* < 0.05). The *p*-values are Bonferroni corrected *p*-values.

### Predictors of FEV_1_ change

Several variables had significant (*p* < 0.2) associations with FEV_1_ change, including: age at initial exam, age at hire, FEV_1_ at initial exam, sex, smoking status, frequent cough, shortness of breath, wheezing, other breathing problems, Department of Energy (DOE) job category, and welding fumes (Table 5). The comorbidity clusters did not have a significant (*p* < 0.436) association with the FEV_1_ change (Table 5). However, several of the comorbid conditions which comprise the clusters were significantly (*p* < 0.2) associated with FEV_1_ change. These included myocardial infarction, coronary artery disease, hypertension, stroke, kidney problems, lung cancer, non-pulmonary cancer, rheumatoid arthritis, anemia, and asthma (Table 5).

**Table 5:** Associations of 1 FEV_1_ change and 1 FEV_1_ decline with several potential predictor variables among the National Supplemental Screening Program participants with chronic obstructive pulmonary at initial exam.

	^1^ **FEV**_**1**_ ** Change**			^1^ **FEV**_**1**_ ** Decline**		
**Characteristics**	**Point estimate**	**95% confidence interval**	***P*-value**	**Odds ratio**	**95% confidence interval**	***P*-value**
**Age at initial exam**	−0.03	−0.04, −0.02	<0.0001	1.05	1.09, 1.07	<0.0001
**Age at hire**	−0.01	−0.02, −0.0004	0.0411	1.01	0.9978, 1.03	0.0938
^1^ **FEV**_**1**_ ** at initial exam**	0.94	0.88, 1.01	<0.0001	0.14	0.11, 0.19	<0.0001
**Sex**			<0.0001			<0.0001
Male	0.70	0.51, 0.89	<0.0001	0.41	0.30, 0.58	<0.0001
Female	*Reference*					
**Smoking status**			0.1620			0.1753
Yes	−0.12,	−0.30, 0.50	0.1620	1.23	0.91, 1.65	0.1753
No	*Reference*					
**Frequent cough**			<0.0001			0.0005
Yes	−0.45	−0.62, −0.28	<0.0001	1.72	1.27, 2.34	0.0005
No	*Reference*					
**Shortness of breath**			<0.0001			<0.0001
Yes	−0.52	−0.68, −0.36	<0.0001	1.99	1.48, 2.66	<0.0001
No	*Reference*					
**Wheezing**			<0.0001			<0.0001
Yes	−0.45	−0.63, −0.28	<0.0001	1.91	1.40, 2.60	<0.0001
No	*Reference*					
**Other breathing problems**			<0.0001			<0.0001
Yes	−0.61	−0.78, −0.43	<0.0001	2.26	1.63, 3.13	<0.0001
No	*Reference*					
^**2**^ **DOE job category**			0.1942			0.2781
Uranium processing facilities	−0.36	−0.75, 0.03	0.0733	1.72	0.85, 3.51	0.1319
Weapons production	−0.11	−0.33, 0.10	0.3074	1.03	0.71, 1.49	0.8792
Science and laboratory facilities	*Reference*					
**Welding fumes exposure**			0.0907			0.0312
Yes	0.14	−0.02, 0.31	0.0907	0.73	0.55, 0.97	0.0312
No	*Reference*					
**Cluster assignment**			0.4357			0.7412
Cluster 2	−0.14	−0.63, 0.35	0.5677	1.23	0.60,	0.5633
Cluster 3	−0.38	−1.05, 0.30	0.2786	1.79	0.48,	0.3791
Cluster 4	−0.70	−1.66, 0.26	0.1577	4.87	0.00, 80331	0.9851
Cluster 1	*Reference*					
**Myocardial infarction**			0.0025			0.0112
Yes	−0.42	−0.69, −0.15	0.0025	1.92	1.16, 3.17	0.0112
No	*Reference*					
**Coronary artery disease**			0.0051			0.0278
Yes	−0.34	−0.58, −0.10	0.0051	1.63	1.05, 2.51	0.0278
No	*Reference*					
**Hypertension**			0.1138			0.0116
Yes	−0.143	−0.32, 0.35	0.1138	1.49	1.09, 2.02	0.0116
No	*Reference*					
**Stroke**			0.0258			0.5535
Yes	−0.37	−0.70, −0.05	0.0258	1.18	0.68, 2.06	0.5535
No	*Reference*					
**Kidney problems**			0.0158			0.0548
Yes	−0.31	−0.56, −0.06	0.0158	1.57	0.99, 2.50	0.0548
No	*Reference*					
**Lung cancer**			0.0663			0.0584
Yes	−0.52	−1.04, −0.004	0.0663	2.80	0.96, 8.14	0.0584
No	*Reference*					
**Non-pulmonary cancer**			0.0666			0.0364
Yes	−0.17	−0.35, 0.01	0.0666	1.40	1.02, 1.92	0.0364
No	*Reference*					
**Rheumatoid arthritis**			0.1468			0.1345
Yes	−0.24	−0.57, 0.09	0.1468	1.57	0.87, 2.86	0.1345
No	*Reference*					
**Anemia**						0.0142
Yes	−0.46	−0.74, −0.19	0.0009	1.87	1.13, 3.08	0.0142
No	*Reference*					
**Asthma**			<0.0001			0.0049
Yes	−0.43	−0.63, −0.23	<0.0001	1.68	1.17, 2.42	0.0049
No	*Reference*					
**Time between exam**	0.01	−0.02, 0.44	0.5281	0.98	0.93, 1.04	0.5686
**Race**			0.6056			0.4745
Non-Black	−0.09	−0.43, 0.25	0.6056	1.24	0.69, 2.22	0.4745
Black	*Reference*					
**Duration of employment**			0.3840			0.1760
5–9 years	−0.09	−0.35, 0.16	0.4698	1.32	0.85, 1.68	0.2130
10–14 years	0.16	−0.14, 0.46	0.2989	0.76	0.46, 2.06	0.2781
15–19 years	0.03	−0.28, 0.31	0.9197	1.33	0.79, 1.25	0.2835
20–24 years	0.24	−0.12, 0.60	0.1942	0.81	0.44, 1.48	0.4876
25–29 years	−0.23	−0.57, 0.12	0.2054	1.59	0.86, 2.97	0.1417
≥ 30 years	0.01	−0.24, 0.26	0.9433	1.31	0.85, 2.02	0.2265
<5 years	*Reference*					
**Cadmium exposure**			0.3749			0.3665
Yes	0.08	−0.09, 0.25	0.3749	0.87	0.65, 1.17	0.3665
No	*Reference*					
**Silica exposure**			0.2828			0.0422
Yes	0.10	−0.07 0.27	0.2828	0.73	0.54, 0.99	0.0422
No	*Reference*					
**Diesel exhaust exposure**			0.5130			0.2289
Yes	0.06	−0.12, 0.25,	0.5130	0.82	0.60, 1.13	0.2289
No	*Reference*					
**Underweight**			0.3901			0.8415
Yes	−0.36	−1.19, 0.46	0.3901	1.16	0.27, 4.89	0.8415
No	*Reference*					
**Overweight or obese**			0.9099			0.1520
Yes	−0.01	−0.20, 0.18	0.9099	1.26	0.92, 1.74	0.1520
No	*Reference*					

**Notes.**

1 Forced expiratory volume in one second.

2 Department of Energy.

Overall, the root mean square errors (RMSE) of the regression random forest models were fairly low across the training (median: 0.85, IQR: 0.83–0.86) and testing (median: 0.84, IQR: 0.81–0.87) datasets. Yet, the amount of variance explained by the model was also low. Only 49.3% (IQR: 48.4%–52.1%) of the variance in the training data and 47.0% in the testing data (IQR: 44.1%–52.4%) were explained by the regression random forest model. At its maximum, the variance explained by the model was 53.8% for the training data and 56.6% for the testing data. However, even with the relatively poor model performance, the variable importance scores still provide interesting insight into which variables are most influential for predicting the FEV_1_ change from initial to rescreen. The FEV_1_ at initial exam (variable importance median: 449.1), age at initial exam (variable importance median: 75.0), age at hire (variable importance median: 68.1), and sex (variable importance median: 47.7) had the highest median variable importance scores (Table 6). The rest of the assessed variables had importance scores less than 20 (Table 6).

**Table 6:** Importance scores for predictors of change in 1 FEV_1_ and 1 FEV_1_ decline for National Supplemental Screening Program participants with chronic obstructive pulmonary at initial exam (*n* = 769) across all imputed datasets (*n* = 20).

	**Predicting** ^1^ **FEV**_**1**_ ** Change** ^3^		**Predicting** ^1^ **FEV**_**1**_ ** Decline** ^4^	
**Characteristic**	**Median**	**IQR**	**Median**	**IQR**
^1^ **FEV**_**1**_ ** at initial exam**	449.1	444.3, 457.5	124.1	121.1, 125.7
**Age at initial exam**	75.0	72.1, 76.9	33.3	31.9, 34.5
**Age at hire**	58.1	57.0, 59.9	28.5	27.6, 29.6
**Sex**				
Male	47.71	45.7, 53.1	12.7	11.2, 14.6
Female	*Reference*			
**Other breathing problems**				
Yes	16.5	14.9, 18.8	4.6	4.0, 5.0
No	*Reference*			
**Frequent cough**				
Yes	12.3	11.2, 13.9	4.2	4.0, 4.4
No	*Reference*			
**Asthma**				
Yes	11.6	9.6, 12.7	3.4	3.1, 3.7
No	*Reference*			
**Shortness of breath**				
Yes	11.5	10.8, 12.9	4.7	4.4, 5.1
No	*Reference*			
**Wheezing**				
Yes	10.0	9.0, 11.2	4.0	3.8, 4.4
No	*Reference*			
**Welding fumes**				
Yes	10.0	9.5, 10.5	5.4	4.6, 5.5
No	*Reference*			
**Smoking status**				
Yes	9.0	8.6, 9.7	5.0	4.8, 5.3
No	*Reference*			
**Non-pulmonary cancer**				
Yes	8.8	8.2, 9.5	3.7	3.5, 3.8
No	*Reference*			
**Hypertension**				
Yes	8.0	7.6, 8.3	3.9	3.8, 4.3
No	*Reference*			
**Anemia**				
Yes	7.6	6.7, 7.9	2.8	2.6, 3.0
No	*Reference*			
**Kidney problems**				
Yes	7.5	6.6, 9.2	N/A	N/A
No	*Reference*			
**Rheumatoid arthritis**				
Yes	6.4	5.9, 7.5	1.9	1.7, 2.2
No	*Reference*			
**Coronary artery disease**				
Yes	5.1	4.7, 5.5	2.6	2.4, 2.8
No	*Reference*			
**Stroke**				
Yes	5.0	4.4, 5.8	N/A	N/A
No	*Reference*			
**Myocardial infarction**				
Yes	4.6	4.3, 4.7	2.7	2.6, 3.1
No	*Reference*			
^2^ **DOE job category**				
Uranium Processing Facilities	3.3	3.0, 3.7	N/A	N/A
Weapons production	7.9	7.4, 8.4	N/A	N/A
Science and laboratory facilities	*Reference*			
**Lung cancer**				
Yes	1.9	1.6, 2.2	0.9	0.8, 1.0
No	*Reference*			
**Silica exposure**				
Yes	N/A	N/A	5.1	5.0, 5.4
No	*Reference*			
**Overweight and obese**				
Yes	N/A	N/A	4.3	4.1, 4.4
No	*Reference*			
**Duration of employment**				
5–9 years	N/A	N/A	3.4	3.2, 3.5
10–14 years	N/A	N/A	3.5	3.2, 3.6
15–19 years	N/A	N/A	4.3	4.0, 4.6
20–24 years	N/A	N/A	1.7	1.6, 1.8
25–29 years	N/A	N/A	2.4	2.1, 2.6
≥ 30 years	N/A	N/A	2.8	2.7, 3.0
<5 years	*Reference*			

**Notes.**

1 Forced expiratory volume in one second.

2 Department of Energy.

3 Variable importance scores are based on regression random forest models.

4 Variable importance scores are based on classification random forest models.

### Predictors of FEV_1_ decline

The following variables had significant (*p* < 0.2) associations with FEV_1_ decline: age at initial exam, age at hire, FEV_1_ at initial exam, sex, smoking status, frequent cough, shortness of breath, wheezing, other breathing problems, welding fume exposure, silica exposure, and duration of employment (Table 5). Similar to the results of the FEV_1_ change models, there was no significant (*p* < 0.741) association between FEV_1_ decline and comorbidity clusters. However, when assessed individually, several comorbid conditions had significant associations with FEV_1_ decline, including: myocardial infarction, coronary artery disease, hypertension, kidney problems, lung cancer, non-pulmonary cancer, anemia, asthma, and overweight or obese, (Table 5).

Unlike the regression random forest models, however, the classification random forest model performed fairly well in predicting FEV_1_ decline. The median classification accuracy across all imputed datasets was 76.8% (IQR: 75.4%–77.8%) in the training data and 76.5% (IQR: 75.0%–78.8%) in the testing data. The model also produced similar levels of performance based on sensitivity and specificity assessment with medians of 78.8% (IQR: 77.8%–79.6%) and 73.5% (IQR: 72.7%–74.8%), respectively, for the training data. In the testing data, the median sensitivity was 82.8% (IQR: 79.4%–85.6%) while the median specificity was 68.7% (IQR: 64.6%–71.7%). Variables which had the highest importance score for predicting FEV_1_ decline were: FEV_1_ at initial exam (median variable importance score: 124.1), age at initial exam (median variable importance score: 33.3), age at hire (median variable importance score: 28.5), and sex (median variable importance score: 12.7) (Table 6). The scores for the remaining variables were all less than 6.0.

## Discussion

This study was designed to identify and describe clusters of COPD comorbidities within an occupational cohort of former US DOE workers. The study also investigated predictors of lung function changes or decline. Study findings may be useful in enhancing health programs screening for comorbidities and for medical management of COPD.

### Comorbidity cluster identification

This study identified four unique clusters of COPD-related comorbidity conditions, which is consistent with the 4–6 comorbidity clusters identified in previous studies (Vanfleteren et al., 2013; Chubachi et al., 2016; Jureviciene et al., 2022; Vikjord et al., 2022; James et al., 2024). In the current study, cluster 1 had lower prevalence proportions of myocardial infarction, coronary artery disease, hypertension, non-pulmonary cancer, and overweight or obesity than all other clusters. This suggests that cluster 1 may represent a cluster of otherwise healthy individuals with COPD, which is consistent with findings from other studies (Vanfleteren et al., 2013; Chubachi et al., 2016; Vikjord et al., 2022; James et al., 2024). In previous studies, a lower general prevalence of all comorbid conditions was significantly associated with less severe COPD outcomes (Vikjord et al., 2022; James et al., 2024). Unfortunately, the severity of long-term COPD outcomes similar to those used in previous studies could not be assessed in this study. Thus, cluster 1 represents a less severe COPD cluster potentially requiring less complicated medical management.

Cluster 2 had a higher prevalence of cardiovascular conditions than cluster 1 while also having a lower prevalence of many non-cardiovascular conditions than clusters 3 and 4. Thus, cluster 2 is a high cardiovascular disease prevalence cluster, which is consistent with findings from many other studies (Vanfleteren et al., 2013; Chubachi et al., 2016; Jureviciene et al., 2022; Vikjord et al., 2022; James et al., 2024). In general, the pathophysiological processes for cardiac function and respiration are interrelated (André et al., 2019). Thus, respiratory dysfunction such as that produced by COPD could lead to cardiac abnormalities, which can ultimately result in worse prognosis when both COPD and cardiovascular disease occur (André et al., 2019; James et al., 2024).

Both clusters 3 and 4 had higher prevalence proportions of most conditions than clusters 1 and 2, which is suggestive of more complicated or severe COPD within these clusters (Vikjord et al., 2022; James et al., 2024). Although not statistically different, the lung cancer prevalence in cluster 3 was double the prevalence in cluster 4, which suggests that cluster 3 may be comprised of individuals with high lung cancer prevalence. The co-occurrence of COPD and lung cancer has been reported by other studies which estimated that 40%–70% of those with lung cancer develop COPD while those with COPD are 2–7 times more likely to develop lung cancer (Qi, Sun & Xiong, 2022; Forder et al., 2023). Although the biological mechanism resulting in the co-occurrences of COPD and lung cancer is unknown, it is thought to be related to a combination of genomic factors, chronic inflammation, and shared risk factors (Qi, Sun & Xiong, 2022; Forder et al., 2023). Specifically, several genetic factors have been identified as possible shared genetic risk factors between the two conditions including Iron Responsive Element Binding Protein 2 (*IREB2*) and Family with Sequence Similarity 13 Member A (*FAM13A*) (Qi, Sun & Xiong, 2022). Regarding shared risk factors, smoking is recognized as a major risk factor for both COPD and lung cancer while other environmental factors such as air pollution are also shared risk factors for both conditions especially among non-smokers (Dubin & Griffin, 2020; Sin et al., 2023).

### Prediction of FEV_1_ change

Although comorbidity clusters were not significant predictors of FEV_1_ change, there were significant associations between FEV_1_ change and multiple comorbid conditions including history of asthma, myocardial infarction, and kidney problems. However, the importance of these comorbid conditions in predicting FEV_1_ change was limited. The biological mechanism driving the co-occurrence of multiple comorbidities and COPD is thought to be multifactorial arising from the interplay of genetic, environmental, and lifestyle factors such as smoking (Fabbri et al., 2023). The reason for the limited predictive importance of the comorbid conditions and the lack of significant association with comorbidity clusters is unclear but might be related to the population studied. This study included individuals with potentially higher exposure to noxious substances, which could also contribute to lung function changes, than the general population (Christenson et al., 2022; Global Initiative for Chronic Obstructive Lung Disease, 2024). Another potential reason is the differences between machine learning and traditional statistical approaches (Boueiz et al., 2022). A study by Boueiz et al. reported similar findings and attributed it to methodological differences between traditional statistical analyses and novel machine learning approaches (Boueiz et al., 2022). Additionally, this study predicted FEV_1_ change over a relatively short time period, which may have contributed to the lack of association between the comorbidity clusters and FEV_1_.

Although comorbidities had limited abilities for predicting FEV_1_ change, several other factors were identified as important predictors. For instance, age at initial exam was one of the strongest predictors of FEV_1_ change in this study, a finding that is consistent with reports from other studies (Tudorache et al., 2017; Thomas et al., 2019; Bae et al., 2024). Across a healthy lifespan, lung function is expected to change, and even decline, with age, most likely resulting from the natural consequences of respiratory aging such as loss of respiratory muscle strength or pulmonary elasticity (Thomas et al., 2019). This notion is often true for COPD as the condition is characterized by accelerated lung function decline (Calverley & Walker, 2023). Age at hire was also of high importance for predicting FEV_1_ change in the current study. However, it is difficult to compare the importance of age at hire to results from other studies as occupational factors are often not included in studies of the general population (Tudorache et al., 2017; Thomas et al., 2019; Boueiz et al., 2022; Bae et al., 2024). Suffice it to say that these findings may be suggestive of the importance occupational factors play in lung function changes especially given that exposure to welding fumes was a significant, albeit weak, predictor in this study.

### Prediction of FEV_1_ decline

The significant association between FEV_1_ decline and cardiovascular disease identified in this study is consistent with reports from other studies (Silvestre et al., 2018; Ramalho & Shah, 2020; Whittaker et al., 2021; Shnoda, Gajjar & Ivanova, 2021; Rosso, Egervall & Elmståhl, 2022; Polman et al., 2024). Lung function impairment can result in cardiovascular disease/events (Ramalho & Shah, 2020), among both individuals with and those without COPD (Shnoda, Gajjar & Ivanova, 2021; Polman et al., 2024). As was the case for FEV_1_ change, cardiovascular disease had limited predictive importance in predicting FEV_1_ decline among those with COPD. By contrast, a study by Whittaker et al. reported no significant association between cardiovascular disease and accelerated FEV_1_ decline among individuals with COPD (Whittaker et al., 2021). The low predictive importance of cardiovascular diseases for predicting FEV_1_ decline may be due to variations in lung trajectories (Calverley & Walker, 2023). Traditionally, accelerated FEV_1_ decline after complete respiratory maturity is a characteristic of COPD (Calverley & Walker, 2023), but a second lung trajectory is also recognized (Lange et al., 2015; Calverley & Walker, 2023). Instead of experiencing accelerated FEV_1_ decline after reaching normal lung capacity, these individuals never fully reach peak respiratory maturity and experience a normal rate of respiratory decline that can also result in COPD (Lange et al., 2015; Calverley & Walker, 2023).

Contrary to reports from other studies (Chen et al., 2020; Perez-Padilla et al., 2017; Thomas et al., 2019; Lee et al., 2022), smoking was not an important predictor of FEV_1_ decline in this study. One possible explanation is the potential role of occupational factors in lung function decline. As previously mentioned, the current study used data from an occupational cohort, whose members may have been exposed to other substances that could affect lung function decline such as welding fumes and silica exposure (Haluza, Moshammer & Hochgatterer, 2014; Barnes et al., 2019; Riccelli et al., 2020). The limited predictive ability of smoking in the current study and the fact that several occupational factors such as welding fumes, silica, and duration of employment were predictors of FEV_1_ decline is suggestive of a possible occupational exposure contribution to lung function decline. Additionally, the pervasiveness of ever smokers within the cohort could also be impacting the predictive ability of the smoking variable. However, the identification of other occupational factors such as age at hire, which was significant predictors of both FEV_1_ decline and FEV_1_ change, further highlights the possible importance of occupational factors in lung function.

### Strengths and limitations

A strength of this study is the application of novel methods in a unique occupational cohort that allowed for the concurrent assessment of occupational factors, demographic characteristics, and medical history. Additionally, by focusing on the relatively short time period for lung function prediction, this study aimed to identify individual characteristics that could be modified before long-term changes occurred.

A limitation of this study is that it relied on a number of self-reported measures including medical history and occupational exposure information, which could be impacted by recall bias (Dohoo, Martin & Stryhn, 2012). Additionally, self-reported asthma was investigated as risk factor for predicting lung function change and decline as a way to account for the irregular obstruction experienced by those with asthma since the data did not capture any other indications of asthmatic symptoms. The study may also be subject to volunteer bias, which is inherent to the screening program as participants must self-select to participate in the National Supplemental Screening Program (NSSP) (Dohoo, Martin & Stryhn, 2012). The potential voluntary bias may also impact the generalizability of results (Dohoo, Martin & Stryhn, 2012; Basagaña et al., 2013). Finally, the current study uses the GOLD criterion as the standard for defining COPD instead of the lower limit of normal standard which was not available in the NSSP data. Additionally, the study did not include bronchodilator reversibility testing, which could help distinguish between COPD, asthma, and asthma-COPD overlap syndrome. This could possibly result in the misclassification of individuals with asthma as COPD patients.

## Conclusions

This study identified clusters of COPD related comorbidities and important predictors of lung function changes and decline within an occupational cohort. Study findings suggest that occupational cohorts not only have different COPD-related comorbidities than the general population but may also have different predictors of lung function changes than the general population. Thus, the findings from this study can be used to target modifiable predictors as well as guide enhanced monitoring efforts.

##  Supplemental Information

10.7717/peerj.20696/supp-1 Supplemental Information 1 R Code used to investigate comorbidity clusters

10.7717/peerj.20696/supp-2 Supplemental Information 2 R Code for Random Forest Model

## Additional Information and Declarations

### Data Availability

The following information was supplied regarding data availability: The Comprehensive Epidemiologic Data Resource is available at: CEDR. (2025). Chronic obstructive pulmonary disease among former United States Department of Energy workers: comorbidities and lung function changes. https://oriseapps.orau.gov/CEDR/search_results.aspx?DataSet=NPH25A01.
